# Estimated financial and human resources requirements for the treatment of malaria in Malawi

**DOI:** 10.1186/1475-2875-6-168

**Published:** 2007-12-19

**Authors:** Adamson S Muula, Emmanuel Rudatsikira, Seter Siziya, Ronald H Mataya

**Affiliations:** 1 Department of Community Health, College of Medicine, University of Malawi, Blantyre, Malawi; 2 Department of Epidemiology, School of Public Health, University of North Carolina at Chapel Hill, North Carolina, USA; 3 Departments of Epidemiology and Biostatistics and Global Health, School of Public Health, Loma Linda University, Loma Linda, California, USA; 4 Department of Community Medicine, School of Medicine, University of Zambia, Lusaka, Zambia; 5 Department of Global Health, School of Public Health, Loma Linda University, California, USA

## Abstract

**Background:**

Malaria fever is a common medical presentation and diagnosis in Malawi. The national malaria policy supports self-diagnosis and self-medication for uncomplicated malaria with first line anti-malaria drugs. While a qualitative appreciation of the burden of malaria on the health system is recognized, there is limited quantitative estimation of the burden malaria exacts on the health system, especially with regard to human resources and financial burden on Malawi.

**Methods:**

The burden of malaria was assessed based on estimated incidence rates for a high endemic country of which Malawi is one. Data on the available human resources and financial resources committed towards malaria from official Malawi government documents and programme reports were obtained. The amount of human and financial resources that would be required to treat 65% or 85% of symptomatic malaria cases as per the Roll Back Malaria partnership and the US President's Malaria Initiative targets.

**Results:**

Based on a malaria incidence rate of 1.4 episodes per year per person it was estimated that there would be 3.71 million symptomatic episodes of malaria among children <5 years of age based on mid-2007 census projections. At 0.59 episodes each year per person there would be 2.13 million episodes in the 5 to 14 year age group and 1.02 million episodes in. There would be 761,848 malaria cases when HIV is not factored in among those 15 years of age or above; this figure rose to 2.2 million when the impact of HIV in increasing malaria incidence was considered. The prevalence of HIV has resulted in 42.3% increase in symptomatic malaria cases. Treating 65% to 85% of cases would result in using 8.9% to 12.2% of the national health budget or 22.2% to 33.2% of the national drug budget. Furthermore, having 65% to 85% of cases treated at a health facility would consume 55.5% to 61.1% of full-time equivalents of all the clinicians registered in the country. While this study's estimated time of 5 and 10 minutes per consultation may differ in actual practice, due to time constraints patients may not be seen for longer consultation in resources limited settings.

**Conclusion:**

Malaria exacts a heavy toll on the health system in Malawi. The national recommendation of self-medication with first-line drug for uncomplicated malaria is justified as there are not enough clinicians to provide clinical care for all cases. The Malawi Ministry of Health's promotion of malaria drug prescription including other lower cadre health workers may be justified.

## Background

Malaria is the leading cause of morbidity and mortality in Malawi. Children under the age of 5 years and pregnant women are especially vulnerable. The Ministry of Health estimates that 40–50 percent of all out-patient health facility attendances are due to malaria [1,2]. It has been reported that an estimated 8 million symptomatic malaria episodes occurs each year in a country of about 13 million people [2].

The Malawi government through the National Malaria Control Programme (NMCP) aims to reduce the public health burden that malaria exacts on the country through improved case management, intermittent presumptive treatment of pregnant women and prevention mostly through the promotion of the use of insecticide-treated bed nets [1]. The treatment for uncomplicated malaria in Malawi has been single dose oral sulphadoxine pyrimethamine (SP) since 1993 [3-5]. Recently however the Malawi Ministry of Health's Policy has changed based on published results [3-5] from SP to a artemisinin-containing combination therapy (ACT), artemether-lafumentrine (coarterm).

While the burden of malaria in terms of clinical outcomes such as deaths, anaemia, prematurity and low birth weight have been described [6-8], the toll that the disease exacts on Malawi's human resources for health and the national health budget has received limited attention. Yet many health systems in developing countries are unable to deliver adequate quality and quantity of health services because of limited human resources for health (HRH) available for service [9-11]. Malawi's health system has experienced severe shortage of HRH from a combination of factors: low output from the training institutions, migration of health professionals and attrition to deaths largely estimated to be from AIDS [12-14].

The main aim of the study was to estimate how much clinician-time that malaria exacts on Malawi's Ministry of Health resources. Secondary aims were to estimate the proportion of finances that anti-malarial medications exact on the country's health budget. Furthermore the study also aimed to compare the estimated whether the Malawi public health sector had adequate human resources to provide treatment to 65 percent or 85 percent of symptomatic malaria as per the Roll Back Malaria partnership and the US President's Malaria Initiative targets [15,16]. Incidence of malaria in Malawi was based on the MARA (Mapping Malaria Risk in Africa) index [17,18].

## Methods

### Population estimates and number of clinicians

The age-specific population estimates from the National Statistical Office which were based on projections from the 1998 Malawi Population and Housing Census [19]. By mid 2007, there were 2,648,694 persons <5 years, 3,612,050 ages 5 to 14 years and 6,925,888 for those aged 15 year or above.

### The incidence of malaria

The incidence of symptomatic malaria by level of transmission intensity and age group was estimated as reported elsewhere [17,18]. As a high malaria endemic area the incidence rate of malaria in Malawi was estimated at 1.4 episodes per-year among children less than 5 years of age, 0.59 episodes in the 5 to 14 years age group and 0.11 episodes per year for those 15 years or older The 2004 Malawi Demographic and Health Survey estimated that 11.8 percent of Malawians ≥ 15 years were infected with HIV [1]. HIV prevalence estimates in children are not known. This then translate to 817,255 persons estimated to be HIV infected in mid-2007.

Incidence rates of malaria among HIV infected adults in Malawi have been estimated at 3.2, 4.9 and 5.4 per 1,000 person-years for those with CD4 count of >400/μL, 200–399/μl and <200/μL [12]. Based on the fact that 11.8% of adult Malawians were estimated to be infected with HIV, the burden of malaria in this group based on population estimated and incidence rates as reported by Patnaik *et al *[20].

The national budget data to enable us assess what proportion of the overall national health budget and drug budget would malaria consume was obtained. Data for the national expenditure was obtained from the Malawi Poverty Reduction Strategy Report [21] and from a report by Kabuluzi [2]. In Malawi, drugs are estimated to consume 30 percent of the total health affairs budget [21]. The estimated medications cost for adult full course was US$ 1.35 and US$ 0.45 for <5 year olds and 5 to 14 year olds [22].

### Number of clinicians in Malawi

There were 610 clinicians (physicians and clinical officers) registered with the Malawi Medical Council in Malawi in 2005 [23]. There were also 485 registered medical assistants. The total clinician pool was therefore 1,095; all clinician cadres included.

## Results

### The estimated case numbers of symptomatic malaria in Malawi

Based on a malaria incidence rate of 1.4 episodes per year, there were an estimated 3,709,572 symptomatic episodes of malaria among children <5 years old in 2005. At 0.59 episodes each year, there were 2,131,110 episodes in 5 to 14 year age group and 761,848 episodes in those aged 15 years or above. An estimated 6,602,530 episodes of malaria would have occurred for the whole population. These estimates were obtained without considering the impact of HIV on malaria.

The estimated number of adult Malawians infected with HIV in the ≥ 15 years age group, based on prevalence of infection at 11.8 percent was 817,255. There is paucity of data that have categorized CD4 count levels of HIV infected Malawians at the population level. It is however generally accepted that 15 percent of HIV infected have CD4 counts less than 200/μL [11]. With 15 percent of 817,255 HIV infected persons with CD4 counts less than 200/μL and assuming the rest have CD4 counts greater than 400/μL, the number of malaria episodes would be 2,222,934 for those will low CD4 counts and 661,975 for those with CD4 counts of >400/μL. The total burden of malaria among adult HIV infected persons would be 2,884,491 compared to 89,898 if HIV was not a factor among this group i.e. a factor of 32 in the presence of HIV. The total number of case in the whole population would then be 9,397,123, an increase of 42.3 percent. The budget and human resources burden that follow were estimated with incidence estimates that factored in HIV's influence in raising the incidence in the 15+ year old group.

### The burden of malaria on the national drug budget

A total US$ 54.3 million (MK 6,412.16 million) was reported to have been spent on health affairs in the 2004/2005 Malawi national budget. The drug budget was US$ 19, 916,101.7. While assessing the cost of treating all cases of malaria would be informative, that would probably be unrealistic. It was therefore perceived that there was need to assess the medication cost of treating 65 percent and 85 percent of all symptomatic cases as outlined in the Roll Back Malaria partnership and the US President's Malaria Initiative targets.

As shown in the tables 1,2,3 and 4, without factoring in the contribution of HIV on malaria, drug costs for treatment of uncomplicated malaria with coartem would consume about 8.9–12.2 percent of the total national budget and about 24.2–33.2 percent of the total drug budget. The proportion of the amount of expenditure differed according to whether only 65 percent or 85 percent of the cases were being treated.

**Table 1:** Burden of malaria treatment on total national health budget at 65% clinical cases treated

**Age group**	**Estimated number of cases**	**Total malaria drug costs in US$**	**Expressed as % of national health affairs budget**
0–4	2,411,222	1,085,050	2.0
0 to 14	1,385,222	623,350	1.1
≥ 15	2,311,958	3,121,143	5.7

Total	6,108,402	4,829,543	8.9

**Table 2:** Burden of malaria treatment on total national health budget at 85% clinical cases treated

**Age group**	**Estimated number of cases**	**Total malaria drug costs in US$**	**Expressed as % of national health affairs budget**
0–4	3,153,136	1,418,911	2.6
0 to 14	1,811,444	815,150	1.5
≥ 15	3,023,330	4,383,285	8.1

Total	7,987,910	6,617,346	12.2

**Table 3:** Burden of malaria treatment on total national drug budget at 65% clinical cases treated

**Age group**	**Estimated number of cases**	**Total malaria drug costs in US$**	**Expressed as % of national drug budget**
0–4	2,411,222	1,085,050	5.4
0 to 14	1,385,222	623,350	3.1
≥ 15	2,311,958	3,121,143	15.7

Total	6,108,402	4,829,543	24.2

**Table 4:** Burden of malaria treatment on total national drug budget at 85% clinical cases treated

**Age group**	**Estimated number of cases**	**Total malaria drug costs in US$**	**Expressed as % of national drug affairs budget**
0–4	3,153,136	1,418,911	7.2
0 to 14	1,811,444	815,150	4.1
≥ 15	3,023,330	4,383,285	22.0

Total	7,987,910	6,617,346	33.2

In 2005–6 financial year, the Malawi government allocated US$ 23,080,000 as total funds for malaria treatment, prevention and control [2]. This allocation came from the country's own national budget, WHO, UNICEF, USAID and the Global Fund. From these funds, Coartem costs for all patients would cost 20.9–28.7 percent of that budget.

### The burden of malaria on the clinician pool

The clinician full time equivalents (FTEs) required to treat 65 percent and 85 percent of symptomatic malaria was assessed based on an assumed patient-clinician contact time of 5 minutes and 10 minutes. In fact Nsimba et al [24] have reported a 3.8 minute consultation of under-five children with fever in Tanzania. Makombe et al [25] have reported that each health professional spends 4.4 work-days each week i.e. a total of 230 days each year providing clinical care. Each health professional would potentially consult 11,040 patients each year at 10 minutes and twice the number at 5 minutes. To treat 65% and 85% of all the cases at 10 minutes per visit would require 553 and 724 clinicians respectively working full time i.e. 55.5 or 66.1 percent of all registered clinicians. The number and proportions of clinicians required reduce by half if a consultation time is reduced to 5 minutes.

## Discussion

It was estimated that about 9.4 million cases of uncomplicated malaria would be expected to occur in Malawi in 2007/08. Comparing age groups 0 to 4 years, 5 to 14 years and those >14 years, the number of cases were highest in the youngest group i.e. about 3.7 million cases. Among the oldest group, incidence increased by a factor of 42.3 percent when the impact of HIV in increasing incidence of malaria was factored in.

Malawi, like many other African health systems, relies heavily on non-physician clinicians [26]. Clinical care is, therefore, provided by medical assistants, clinical officers and physicians. It was estimated that 55.5 to 66.1 percent of all registered clinicians would be required to treat 65 percent to 85 percent of the cases of uncomplicated symptomatic malaria in the country. While it is currently unrealistic that all these cases would present for care, the estimate still demonstrates that malaria is a large public health problem in Malawi. Probably a major finding of the current assessment is that both the Roll Back Malaria and the US President's Malaria Initiative targets are unlikely to be achieved in Malawi for the treatment of patients by medical assistants, clinical officers and medical doctors.

The Ministry of Health in Malawi promotes the mobilization of community health workers and lower cadre health professionals for the management of malaria [22]. While such recommendations are certainly realistic, they may contribute to the development of antimalarial drug resistance due to inappropriate use of medications by limited trained health workers.

The burden that malaria would exact on the national health affairs budget is large. However in practice, this is not the case as patients self-medicate at home from drugs obtained from various sources including grocery stores and the private sector [27]. Also health facilities do not always stock adequate medications all the time, so the actual expense may be lower than what has been estimated in the study. A recent survey of health facilities in Lilongwe district, capital district of Malawi by Lufesi et al found that the government-run Central Medical Stores was only able to satisfy 63 percent of antimalarial drug orders [28]. Some health facilities had no antimalarial on the survey at some periods. This study had several limitations. It was estimated that all symptomatic malaria episodes would be effectively treated at the first presentation. Some cases would have to be re-treated with a second line anti-malarial drug. It has also been previously reported that in high endemic areas HIV increases the likelihood of malaria to become severe [29]. With an adult HIV prevalence of 11.8 percent, many cases of malaria in this age group have potential to becoming severe and therefore not amenable to first line treatment regimen.

Furthermore, we assessed the clinician time requirements based at 5 and 10 minutes consultations with patients. This assumption of how much time may be committed was based on the authors' experience in managing patients with malaria in Africa. Rowe et al [30] have reported that 70% of clinicians in their study of malaria management spent not more than 9 minutes per patient consultation. In fact a third of their clinicians spent less than 6 minutes per consult. This should not be seen as an endorsement by the authors as to how much time ought to be spent by clinicians on a single patient, but rather an attempt to justify the current study's estimate of how much time clinician may be actually devoting to care for malaria patients.

The challenges experienced by the Central Medical Stores in ensuring transparency of resources along the chain of drug supply, distribution and eventual use have been described by Lufesi et al [28]. Wastage is a constant threat within the public sector in Malawi. Finally, while only anti-malarial drug expenses were estimated, many patients who present with malaria fever, myalgia, athralgia and other symptoms will also likely be prescribed analgesics; thus an added medication cost. These estimates have been restricted to financial resources within the Ministry of Health and HRH within the whole sector. The financial expenses of the medication costs within the private sector have not been estimated. However, the findings of the study clearly highlight how much resources the country as a whole, requires to treat malaria.

## Conclusion

The financial and human resource burden let alone the human suffering caused by malaria cannot be underestimated especially in a country where the per capita spending on health is only $12, Community based preventive approaches such as the use of insecticide treated bed nets, proper drainage of stagnant water, intermittent preventive therapy of pregnant women and use of community "pharmacies" e.g. grocery stores as distribution centers for malaria drugs will be less costly. As the main provider of healthcare, government needs to study disaggregated data (qualitative and quantitative) pertaining to vertical programmes such as TB, HIV and malaria. This will help health planners and policy makers in formulating policies to improve the quality of services and efficient use of the limited resources the country has.

## Abbreviations

HIV: human immunodeficiency virus;

SP: Sulphadoxine pyrimethamine;

UNICEF: United Nations Children's Fund;

USAID: United States Agency for International Development;

US$: United States dollar;

WHO: World Health Organization.

## Competing interests

The author(s) declare that they have no competing interests.

## Authors' contributions

ASM: designed the study, collected data and participated in drafting the manuscript.

ER: participated in the interpretation of the findings and drafting of manuscript.

SS: participated in the interpretation of the findings and drafting of manuscript

RHM: participated in the interpretation of the findings and drafting of manuscript
